# Pharmacological Validation of an Inward-Rectifier Potassium (Kir) Channel as an Insecticide Target in the Yellow Fever Mosquito *Aedes aegypti*

**DOI:** 10.1371/journal.pone.0100700

**Published:** 2014-06-24

**Authors:** Matthew F. Rouhier, Rene Raphemot, Jerod S. Denton, Peter M. Piermarini

**Affiliations:** 1 Department of Entomology, Ohio Agricultural Research and Development Center, The Ohio State University, Wooster, Ohio, United States of America; 2 Department of Anesthesiology, Vanderbilt University School of Medicine, Nashville, Tennessee, United States of America; 3 Department of Pharmacology, Vanderbilt University School of Medicine, Nashville, Tennessee, United States of America; 4 Institute of Chemical Biology, Vanderbilt University School of Medicine, Nashville, Tennessee, United States of America; 5 Institute for Global Health, Vanderbilt University School of Medicine, Nashville, Tennessee, United States of America; New Mexico State University, United States of America

## Abstract

Mosquitoes are important disease vectors that transmit a wide variety of pathogens to humans, including those that cause malaria and dengue fever. Insecticides have traditionally been deployed to control populations of disease-causing mosquitoes, but the emergence of insecticide resistance has severely limited the number of active compounds that are used against mosquitoes. Thus, to improve the control of resistant mosquitoes there is a need to identify new insecticide targets and active compounds for insecticide development. Recently we demonstrated that inward rectifier potassium (Kir) channels and small molecule inhibitors of Kir channels offer promising new molecular targets and active compounds, respectively, for insecticide development. Here we provide pharmacological validation of a specific mosquito Kir channel (*Ae*Kir1) in the yellow fever mosquito *Aedes aegypti*. We show that VU590, a small-molecule inhibitor of mammalian Kir1.1 and Kir7.1 channels, potently inhibits *Ae*Kir1 but not another mosquito Kir channel (*Ae*Kir2B) in vitro. Moreover, we show that a previously identified inhibitor of *Ae*Kir1 (VU573) elicits an unexpected agonistic effect on *Ae*Kir2B in vitro. Injection of VU590 into the hemolymph of adult female mosquitoes significantly inhibits their capacity to excrete urine and kills them within 24 h, suggesting a mechanism of action on the excretory system. Importantly, a structurally-related VU590 analog (VU608), which weakly blocks *Ae*Kir1 in vitro, has no significant effects on their excretory capacity and does not kill mosquitoes. These observations suggest that the toxic effects of VU590 are associated with its inhibition of *Ae*Kir1.

## Introduction

Adult female mosquitoes are vectors of pathogens that are transmitted during blood feeding to humans and other vertebrates. The yellow fever mosquito *Aedes aegypti* and other Culicine species are vectors of viruses that cause chikungunya, dengue, West Nile, and yellow fevers in humans and/or animals, while the malaria mosquito *Anopheles gambiae* and other Anopheline species are vectors of protozoans that cause malaria in humans and animals. Notably, nearly half of the world's population is at risk of acquiring dengue fever or malaria [1], [2], and chikungunya fever has rapidly spread within the last 10 years from its historical range of Africa and Asia to Europe [3], [4] and the Caribbean islands (www.cdc.gov).

One common strategy that is used to limit the spread of mosquito-borne diseases is to control populations of the mosquito vectors with insecticides. However, such vector control efforts are being compromised by the emergence of insecticide resistance in mosquito populations, thereby making conventional insecticides (e.g., DDT, pyrethroids) ineffective [5], [6]. Thus, the 1) identification of new molecular and physiological targets in mosquitoes, and 2) discovery of active compounds against mosquitoes, are critical to improve vector control efforts [7], [8].

Our group has recently begun to explore inward rectifier K^+^ (Kir) channels in the excretory system of mosquitoes as novel molecular and physiological targets for insecticide development [9]. We have shown that the genome of the yellow fever mosquito *A. aegypti* possesses five genes encoding Kir channel subunits (*Ae*Kir1, *Ae*Kir2A, *Ae*Kir2B, *Ae*Kir2B', and *Ae*Kir3) that exhibit tissue-specific expression patterns in adult females [10], [11]. The renal (Malpighian) tubules primarily express *Ae*Kir1, *Ae*Kir2B, and *Ae*Kir3, where one or more of these channels are considered important mechanisms for the transepithelial secretion of K^+^ and fluid [10]. The hindgut primarily expresses *Ae*Kir2A and *Ae*Kir2B where these channels may contribute to the reabsorption of K^+^ and/or water [11]. Furthermore, we have shown that a small molecule inhibitor of mammalian Kir channels (VU573) inhibits the *Ae*Kir1 channel in vitro and incapacitates adult female mosquitoes at least in part by disrupting their renal excretory functions and hemolymph K^+^ homeostasis [9]. Thus, Kir channels appear to play vital physiological roles in mosquitoes, which make them potentially attractive targets for the development of new insecticides.

Here we aim to further validate the *Ae*Kir1 channel as an insecticide target. We show that a mammalian Kir channel inhibitor (VU590), which is structurally unrelated to VU573, inhibits *Ae*Kir1 in vitro with a greater potency than VU573 and does not affect the activity of *Ae*Kir2B. Injection of VU590 into the hemolymph of adult female mosquitoes disrupts their excretory capacity and kills them within 24 h. Our results validate 1) *Ae*Kir1 as an insecticide target, and 2) small molecule modulators of Kir channels as new active compounds in the development of insecticides against mosquitoes.

## Materials and Methods

### Chemical reagents

The synthesis of VU590, VU573, and VU342 are described in detail elsewhere [12], [13]. VU608 was provided by the Vanderbilt Chemical Synthesis Core (https://medschool.vanderbilt.edu/syncore/).

### Expression vectors and sub-cloning

The pcDNA/TO expression-vector construct (for HEK293 cell studies) containing the open-reading frame of *Ae*Kir1, and the pGH19 plasmid constructs (for *Xenopus* oocyte studies) containing the open-reading frames of *Ae*Kir1 and *Ae*Kir2B, are described elsewhere [9], [10].

### Stable cell line generation and thallium flux assays

The stable monoclonal cell line (T-REx-HEK293 cells) expressing *Ae*Kir1 was generated in a previous study [9]. In brief, these cells were loaded with Thallos-AM (TEFlabs, Austin, TX), which is a Tl^+^-sensitive fluorescent dye, and plated in black-wall and clear-bottom 384-well BD PureCoat amine-coated plates (BD, Bedford, MA), as described previously [9]. All plates were loaded onto a kinetic imaging plate reader (FDSS 6000; Hamamatsu Corporation, Bridgewater, NJ), and the fluorescence recordings were made at room temperature (20–23°C). After the appropriate baseline readings were taken (10 images at 1 Hz; excitation, 470±20 nm; emission, 540±30 nm), 20 µl of the small molecules were added and 50 images were taken at 1 Hz. Twenty minutes after addition of the small molecules the baseline readings were measured for 10 s, 10 µl of Tl^+^ stimulus buffer was added to each well, and an additional 240 images were taken at 1 Hz.

### Heterologous expression and electrophysiology in *Xenopus* oocytes

Capped RNA (cRNA) encoding *Ae*Kir1 or *Ae*Kir2B was synthesized as described previously [10]. Defolliculated *Xenopus laevis* oocytes (Ecocyte Bioscience, Asutin, TX) were injected with 10 ng of *Ae*Kir1 or *Ae*Kir2B cRNA and cultured for 3–7 days at 18°C in OR3 media [14], [15].

All electrophysiological experiments were performed at room temperature as described previously [10]. The compositions of the solutions used are shown in Table 1. When needed, VU590 or VU573 was dissolved in solution *III* to a final concentration of 50 µM (0.05% DMSO). All solutions were delivered by gravity to a RC-3Z oocyte chamber (Warner Instruments, Hamden, CT) via polyethylene tubing at a flow rate of ∼2 ml/min. Solution changes were made with a Rheodyne Teflon 8-way Rotary valve (Model 5012, Rheodyne, Rohnert Park, CA).

**Table 1:** Compositions (in mM) of solutions used in oocyte electrophysiology.

Solution #	*I*	*II*	*III*
NaCl	96	88.5	88.5
NMDG-Cl	0	9.5	0
KCl	2	0.5	10
MgCl_2_	1.0	1.0	1.0
CaCl_2_	1.8	1.8	1.8
HEPES	5	5	5

The pH of all solutions was adjusted to 7.5 with NMDG-OH.

The osmolality of each solution was verified to be 190 mOsm kg^−1^ H_2_O (± 5 mOsm kg^−1^ H_2_O) by vapor pressure osmometry.

NMDG  =  N-methyl-D-glucamine.

For a given experiment, an oocyte was transferred to the holding chamber under superfusion with solution *I* and then impaled with two conventional-glass microelectrodes backfilled with 3 M KCl (resistances of 0.5–1.5 MΩ). One electrode measured membrane potential (V_m_) and the other measured whole-cell membrane current (I_m_). Each microelectrode was bridged to an OC-725 oocyte clamp (Warner Instruments) under the digital control of the Clampex module of pCLAMP software (version 10, Molecular Devices, Sunnyvale, CA).

Current-voltage (I–V) relationships of oocytes were acquired by first clamping an oocyte near its spontaneous V_m_ and then initiating the voltage-stepping protocol via the Clampex module of pCLAMP. In brief, the protocol consists of 20 mV steps from −140 mV to + 40 mV (100 ms each) [10]. The voltage clamp was then turned off and a new solution was superfused through the chamber. Once the oocyte reached a new steady-state V_m_ (∼90 s) the I–V relationship of the oocyte was acquired again as described above. All V_m_ and I_m_ values were recorded by a Digidata 1440A Data Acquisition System (Molecular Devices) and the Clampex module of pCLAMP. The I–V plots were generated with the Clampfit module of pCLAMP.

To evaluate the modulation of Kir activity by the pharmacological compounds, we focused on the maximal inward currents elicited by the voltage-stepping protocol, which occur at a voltage of −140 mV. The background, inward currents in solution *II* (i.e., low K^+^) were subtracted from those in 1) solution *III* (i.e., elevated K^+^) to calculate the total inward current for an oocyte before exposure to VU590 or VU573 (i.e., I_A_), and 2) solution *III* with VU590 or VU573 to calculate the inward current after exposure to a small molecule (i.e., I_B_). The percent change of the inward current was calculated by subtracting I_B_ from I_A_ and then dividing by I_A_. Inhibition and activation are represented as negative and positive percent changes, respectively.

### Mosquito colony

Mosquito eggs were obtained from the MR4 as part of the BEI Resource Repository, NIAID, NIH (deposited by M.Q. Benedict; *Aedes aegypti* LVP-IB12, MRA-735). Mosquitoes were raised to adults as described previously [10] and fed on 10% sucrose ad libitum. Only female mosquitoes 3–10 days post emergence were used in experiments.

### Mosquito toxicology experiments

Mosquitoes were immobilized on ice and then injected with 69 nl of fluid using a needle (pulled from a glass capillary tube) attached to a nanoliter injecting device (Nanoject II, Drummond Scientific Company, Broomall, PA). The injection solution was a potassium-enriched, phosphate-buffered saline (K^+^-PBS; see compositions below) containing the following solvents, which were necessary to maintain the small molecules in solution: 15% DMSO, 1% β-cyclodextran (Acros Organic, Fair Lawn, NJ), and 0.1% Solutol (BASF, Florham Park, NJ). In some experiments, the K^+^-PBS (K^+^-PBS_50_) consisted of the following in mM: 92.2 NaCl, 47.5 KCl, 10 Na_2_HPO_4_, and 2 KH_2_PO4 (pH 7.5). In other experiments, the K^+^-PBS (K^+^-PBS_75_) consisted of the following in mM: 65.9 NaCl, 73.9 KCl, 10 Na_2_HPO_4_, and 2 KH_2_PO_4_ (pH 7.5). For each trial, a total of 10 mosquitoes were injected for a given dose or treatment and placed immediately in small cages (10 mosquitoes per cage) with free access to 10% sucrose. The cages were kept in an environmentally-controlled rearing chamber (28°C, 80% relative humidity, 12 h:12 h light:dark). The mosquitoes were observed 24 h after injection.

### Mosquito excretion experiments

The excretory capacity of intact mosquitoes (*A. aegypti*) was measured after delivering a volume load to the hemolymph, using a modification of a previously described method [9]. In brief, after immobilizing mosquitoes on ice they were injected as described above with 900 nl of fluid (100 nl/s). The injected fluid was K^+^-PBS_50_ containing 1.15% DMSO, 0.077% β-cyclodextran, and 0.008% Solutol. When present, the small molecules (VU590, VU608, VU573, or VU342) were dissolved at 0.77 mM. After injection, the mosquitoes were placed immediately in a graduated, packed-cell volume tube (MidSci, St. Louis, MO; 5 mosquitoes per tube) at 28°C.

In a previous study [9], the mosquitoes remained in a single tube for 2 h and were then removed from the tubes with forceps. The excreted urine was centrifuged into the graduated column of the tube for measurement and the total excreted volume after 2 h was measured. In the present study, preliminary experiments were conducted to better resolve the time-course of urine excretion. That is, mosquitoes were transferred to a new tube every 30 minutes and the amount of urine excreted was measured. As shown in Figure S1, ∼95% of the total volume excreted by the mosquitoes in 120 min is voided within the first 60 min. Thus, we used a 60 min end point for the excretion measurements in the present study.

### Statistical analyses

#### Tl^+^-flux assays

Procedures for analysis of data are described in detail elsewhere [9]. In brief, the slope of the fluorescence increase, between 5 s and 15 s after Tl^+^ addition was calculated for each well, and corrected for any baseline waveforms generated in the presence of vehicle controls. The data were then plotted in Prism (GraphPad Software, San Diego, CA) to generate concentration-response curves; potencies (IC_50_) were calculated from non-linear curve fits using a four parameter logistic equation.

#### Oocyte electrophysiology

To determine whether a potential inhibition or activation of inward current by a small molecule was statistically significant, the percent inhibition values were first transformed (arcsine) and then a one-sample t test with a hypothetical value of ‘0.0’ was performed using Prism (Graphpad Software).

#### Mosquito toxicology and urine excretion

Prism (Graphpad Software) was used to generate a dose-response curve for the toxicity of VU590; the doses (x-axis) were first log transformed and then a non-linear curve was fitted to the mortality data using the ‘log (agonist) vs. response – four parameter variable slope’ algorithm to calculate potency (LD_50_). To compare 1) the toxic effects among the vehicle, VU590, and VU608 treatments, 2) the excretory capacity among the vehicle, VU590, and VU608 treatments, and 3) the excretory capacity among the vehicle, VU573, and VU342 treatments, one-way ANOVAs were performed with Newman-Keuls posttests.

## Results

### Pharmacology of VU590, VU608, and VU573 in vitro

**Figure 1:**
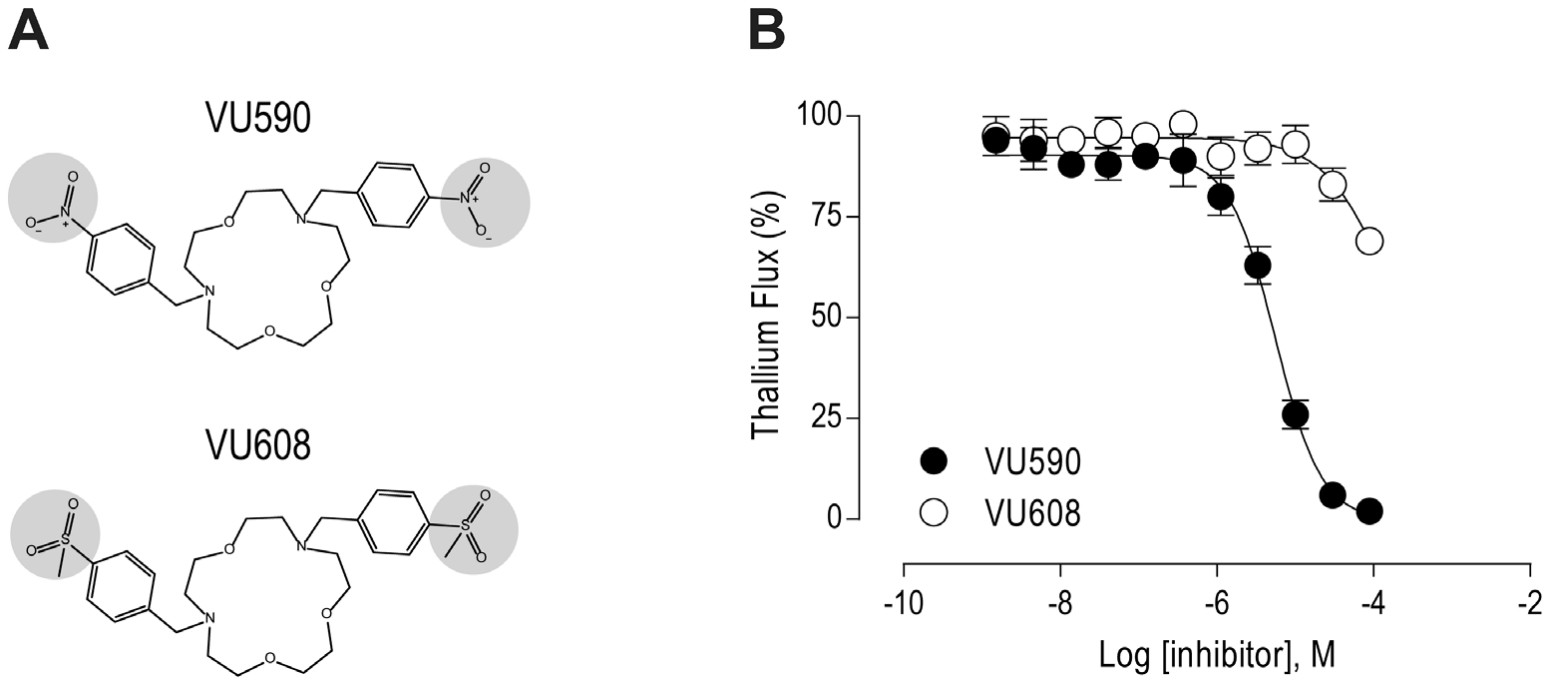
Effects of VU590 and VU608 on *Ae*Kir1 channels expressed heterologously in TREx-HEK293 cells. (A) Chemical structures of the *Ae*Kir1 inhibitor VU590 and its ‘inactive’ analog VU608. Gray shading highlights the chemical differences between the molecules. (B) Concentration-response curves of VU590 (filled circles) and VU608 (open circles) derived from Tl^+^-flux assays. Values are means ± SEM. *n* = 2 independent experiments, each performed in triplicate. The calculated IC_50_ values for VU590 and VU608 are 5.6 µM (95% CI: 4.3–7.2 µM) and >100 µM, respectively.

In a previous study, we developed a fluorescence-based, high-throughput assay of *Ae*Kir1 activity [9]; this so-called ‘Tl^+^-flux’ assay takes advantage of the ability of Tl^+^ to permeate *Ae*Kir1 channels and allows for the rapid identification and pharmacological characterization of small-molecule modulators of Kir channel activity [12]. Here we use the Tl^+^-flux assay to characterize the potential inhibition of *Ae*Kir1 by VU590 (Figure 1A). As shown in Figure 1B, applying VU590 to *Ae*Kir1-expressing HEK293 cells blocks the Tl^+^-flux in a dose-dependent manner with an IC_50_ of 5.6 µM.

Previous medicinal chemistry efforts around the VU590 scaffold to improve its selectivity for human Kir1.1 vs. human Kir7.1 resulted in a compound that shares the same macrocycle core as VU590, but has two (methylsulfonyl)benzyl groups in place of two nitrobenzyl groups (Chauder and Denton, unpublished data); we refer to this compound as VU608 (Figure 1A). As shown in Figure 1B, VU608 exhibits a much weaker block of the *Ae*Kir1-mediated Tl^+^-flux (IC_50_>100 µM) than VU590. Thus, we use VU608 as a small-molecule negative control in experiments with mosquitoes (see ‘Effects of VU590 and VU608 on mosquitoes’ below).

To further characterize the pharmacology of VU590 in vitro, we examined its effects on another mosquito Kir channel, *Ae*Kir2B. Efforts to functionally express *Ae*Kir2B in HEK293 cells were not successful (Raphemot and Denton, data not shown). However, we have previously demonstrated that *Ae*Kir2B forms constitutively active Kir channels when expressed in *Xenopus* oocytes [10]. Thus, we evaluated the effects of VU590 on the electrophysiology of oocytes expressing *Ae*Kir2B (*Ae*Kir2B oocytes) using two-electrode voltage clamping; *Ae*Kir1 oocytes served as positive controls.

**Figure 2:**
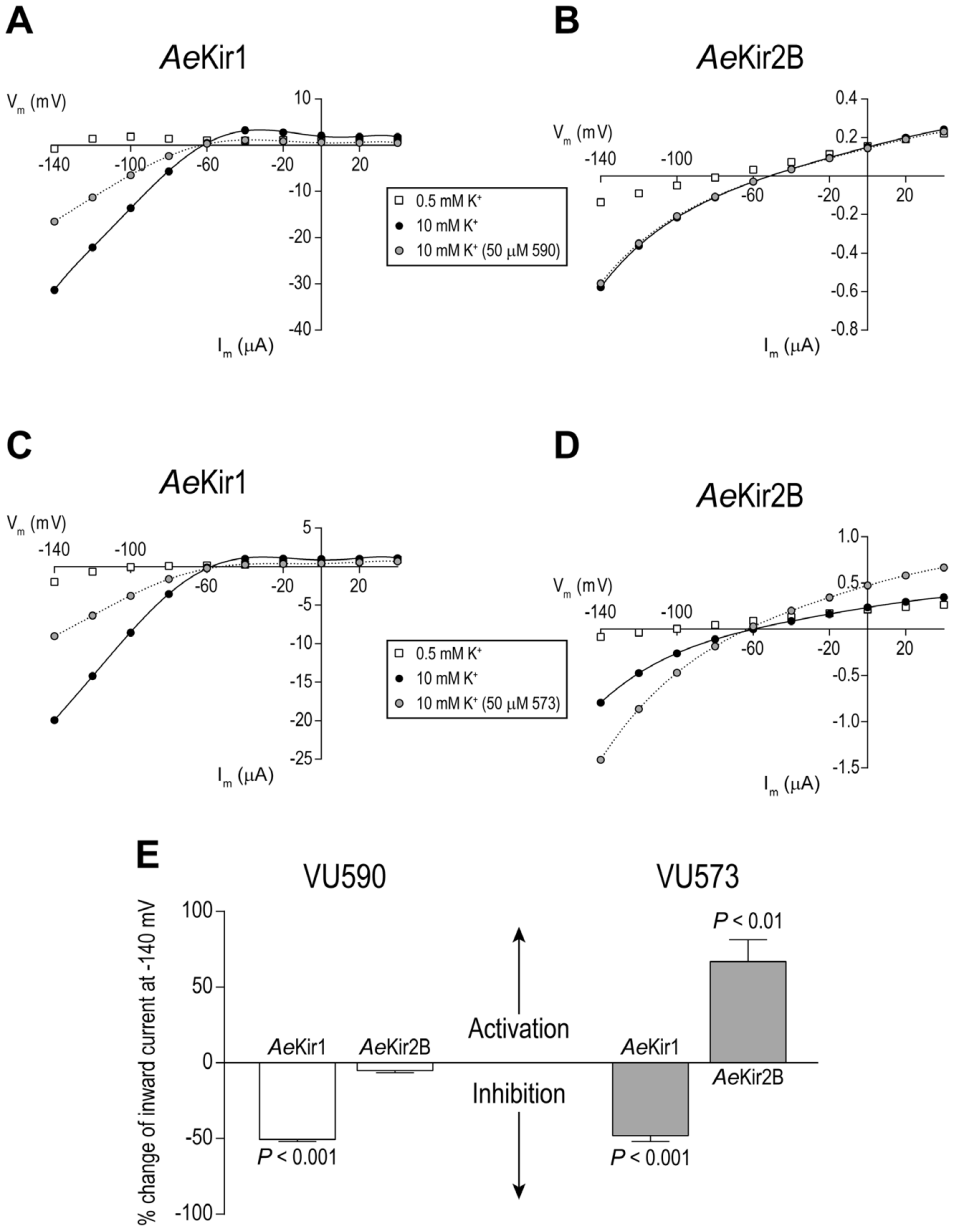
Effects of VU590 and VU573 on *Ae*Kir1 and *Ae*Kir2B channels expressed heterologously in *Xenopus* oocytes. Current-voltage (I–V) relationships of representative *Ae*Kir1 (A, C) and *Ae*Kir2B (B, D) oocytes bathed consecutively in solutions containing 0.5 mM K^+^ (open boxes), 10 mM K^+^ (filled circles), and 10 mM K^+^ + 50 µM of a small molecule (gray circles). The small molecule is VU590 in panels ‘A’ and ‘B’, and VU573 in panels ‘C’ and ‘D’. (E) Summary of the percent changes of inward currents at −140 mV in *Ae*Kir1 and *Ae*Kir2B oocytes elicited by VU590 and VU573. Positive and negative percent changes indicate activation and inhibition, respectively. *P* values indicate significant activation or inhibition as determined by a one sample t test (on arcsine transformed values). Values are non-transformed means ± SEM. For VU590 experiments, *n* = 3 oocytes each for *Ae*Kir1 and *Ae*Kir2B. For VU573 experiments, *n* = 5 and 8 oocytes each for *Ae*Kir1 and *Ae*Kir2B, respectively.

Figure 2A shows the current-voltage (I–V) relationship of a representative *Ae*Kir1 oocyte; the oocyte mediates robust inward currents at hyperpolarizing voltages in the presence of elevated extracellular K^+^ (black circles in Figure 2A). Consistent with the results of Figure 1, addition of VU590 (50 µM) to the extracellular bath blocks the inward K^+^-currents (gray circles in Figure 2A). Figure 2B shows the I–V relationship of a representative *Ae*Kir2B oocyte. Consistent with a previous study [10], the *Ae*Kir2B oocyte mediates weak inward-rectifying K^+^ currents compared to the *Ae*Kir1 oocyte (Figure 2B). The addition of VU590 (50 µM) has no detectable effects on the inward K^+^-currents mediated by the *Ae*Kir2B oocytes (gray circles in Figure 2B).

Given the selective inhibition of *Ae*Kir1 vs. *Ae*Kir2B by VU590, we decided to evaluate the effects of another small-molecule inhibitor of *Ae*Kir1 (VU573) on *Ae*Kir2B oocytes, again using *Ae*Kir1 oocytes as positive controls. Consistent with our previous study [9], VU573 (50 µM) blocks the inward K^+^-currents mediated by *Ae*Kir1 oocytes (gray circles in Figure 2C). However, to our surprise, VU573 (50 µM) activates the inward currents mediated by *Ae*Kir2B oocytes (gray circles in Figure 2D).

Figure 2E summarizes the modulation of inward K^+^ currents by VU590 and VU573 in the *Ae*Kir1 and *Ae*Kir2B oocytes. Whereas VU590 significantly blocks ∼50% of the *Ae*Kir1-mediated K^+^-currents, it does not affect the *Ae*Kir2B-mediated K^+^-currents. Similar to VU590, VU573 significantly blocks ∼50% of the *Ae*Kir1-mediated K^+^-currents, but in contrast to VU590, VU573 significantly activates the *Ae*Kir2B mediated K^+^-currents by over 50%. Thus, VU590 is a selective inhibitor of *Ae*Kir1, whereas VU573 is an inhibitor of *Ae*Kir1 and an agonist of *Ae*Kir2B.

### Effects of VU590 and VU608 on mosquitoes

**Figure 3:**
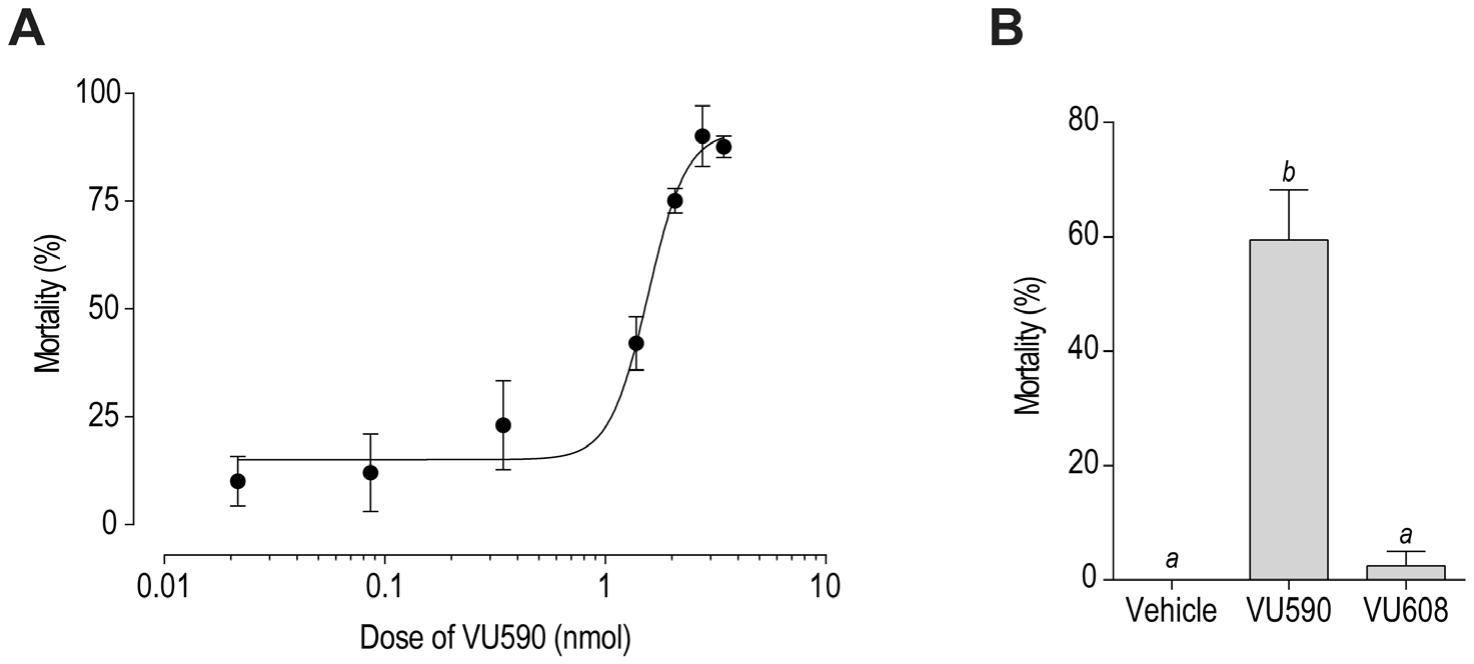
Effects of VU590 on the survival of adult female mosquitoes (*A. aegypti*). (A) Dose-response curve of the toxic effects of VU590 on mosquitoes (R^2^ = 0.87). Mortality was assessed 24 h after injecting the hemolymph with 69 nl of the vehicle (K^+^-PBS_50_ with 15% DMSO, 1% β-cyclodextran, and 0.1% Solutol) containing appropriate concentrations of VU590 to deliver the doses indicated. The calculated LD_50_ is 1.56 nmol (95% CI: 1.29–1.88 nmol). Values are means ± SEM; *n* = 4 trials of 10 mosquitoes per dose. (B) Comparison of the toxic effects of the vehicle, VU590, and VU608. Mortality was assessed 24 h after injecting the hemolymph with the vehicle (K^+^-PBS_75_ with 15% DMSO, 1% β-cyclodextran, and 0.1% Solutol) or the vehicle containing a small molecule (2.8 nmol). Values are means ± SEM; *n* = 4 trials of 10 mosquitoes. Lower-case letters indicate statistical categorization of the means as determined by a one-way ANOVA with a Newman-Keuls posttest (*P*<0.05).

To determine if VU590 perturbs mosquito behavior and physiology, we injected the compound into the hemolymph of adult female mosquitoes. As shown in Figure 3A, injection of VU590 kills mosquitoes within 24 h in a dose-dependent manner with a LD_50_ of 1.56 nmol per female. Obvious sub-lethal effects of VU590 on the behavior of mosquitoes, such as the impairment of flight, were not noticeable.

We next aimed to determine whether the toxic effects of VU590 were correlated with its block of *Ae*Kir1 by injecting mosquitoes with the inactive analog VU608. As shown in Figure 3B, injection of VU590 (2.2 nmol) again significantly increases the mortality of mosquitoes within 24 h compared to those injected with the vehicle, whereas the injection of VU608 does not. Notably in these experiments, ∼17% of the mosquitoes killed by VU590 exhibited extreme abdominal bloating similar to that observed in a previous study with VU573 [9], which suggests that VU590 disrupts fluid volume homeostasis in mosquitoes.

### Effects of VU590 on the excretory capacity of adult female mosquitoes

To determine if the lethal effects of VU590 are associated with a disruption to osmoregulation, we evaluated its effects on the excretory capacity of adult female mosquitoes. In brief, mosquitoes were injected with 900 nl of a phosphate-buffered saline into their hemolymph to introduce a stress to salt and water balance; the amount of urine excreted within the next hour was measured using an approach modified from a previous study [9].

**Figure 4:**
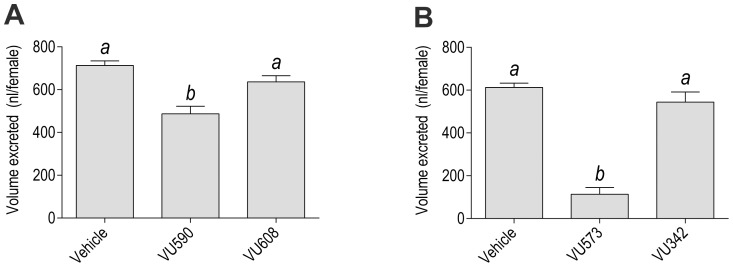
Effects of VU590, VU608, VU573, and VU342 on the in vivo excretory capacity of adult female mosquitoes (*A. aegypti*). (A) Amount of urine excreted by mosquitoes 1 h after injection with 900 nl of the vehicle (K^+^-PBS_50_ containing 1.8% DMSO, 0.077% β-cyclodextrane, and 0.008% Solutol), the vehicle containing VU590 (0.77 mM), or the vehicle containing VU608 (0.77 mM). Values are means ± SEM; *n* = 11 trials of 5 mosquitoes per treatment. Lower-case letters indicate statistical categorization of the means as determined by a one-way ANOVA with a Newman-Keuls posttest (*P*<0.05). (B) Same as ‘A’, but with VU573 and VU342. *n* = 9 trials of 5 mosquitoes per treatment.

Figure 4A shows the volume of urine excreted by mosquitoes 1 h after injection with the vehicle, VU590 (0.77 mM), or VU608 (0.77 mM). Compared to injection of the vehicle, VU590 significantly reduces the amount of urine that mosquitoes excrete by ∼32%, whereas VU608 does not. To put this inhibition in perspective, we also examined the effects of VU573 on urine excretion. Consistent with our previous study [9], VU573 (0.77 mM) significantly reduces the amount of urine that mosquitoes excrete by ∼82% compared to the vehicle, whereas VU342 (an inactive analog of VU573; 0.77 mM) does not (Figure 4B). Thus, VU590 reduces the excretory capacity of mosquitoes, but it is not as effective as VU573.

## Discussion

### VU590 is a selective inhibitor of *Ae*Kir1 compared to VU573

Both VU590 and VU573 are structurally unrelated compounds that were discovered in a high-throughput screen for small-molecule modulators of mammalian Kir1.1 function [12], [13]. VU590 blocks mammalian Kir1.1 and Kir7.1 channels with < 1 µM and < 10 µM potencies, respectively, but does not have detectable effects on mammalian Kir2.1 and Kir4.1 channels [12]. Electrophysiological studies of VU590 and a structurally-derived analog (VU591) on mammalian Kir1.1 suggest that these compounds block the intracellular pore of the channel [12], [16]. In contrast, VU573 blocks Kir3.X, Kir2.3, and Kir7.1 channels with < 10 µM potency, but also blocks Kir1.1 and Kir2.1 channels with > 10 µM potency [13]. The mechanism of inhibition of VU573 on mammalian Kir channels is presently unknown, but preliminary studies suggest that it is not a pore blocker like VU590 (Raphemot and Denton, unpublished observations).

Given the selective nature of VU590 within mammalian Kir channels, we wondered how well it would inhibit *Ae*Kir1 compared to the more promiscuous VU573. The results of the present study demonstrate that VU590 not only blocks *Ae*Kir1 (Figures 1 and 2), but does so with over a 2.5-times greater potency (IC_50_ = 5.6 µM) than VU573 (IC_50_ = 15 µM;[9]), as assessed by the Tl^+^-flux assay. VU590 is further distinguished from VU573 by its selectivity for *Ae*Kir1 vs. *Ae*Kir2B. That is, VU590 blocks *Ae*Kir1 and is without effect on *Ae*Kir2B (Figure 2E), whereas VU573 inhibits *Ae*Kir1 and activates *Ae*Kir2B (Figure 2E). The selectivity of VU590 for *Ae*Kir1 makes it a useful pharmacological tool for dissecting the contributions of Kir1 channels in mosquito physiology, especially in the renal (Malpighian) tubules which express at least 3 mRNAs encoding Kir channel subunits: *Ae*Kir1, *Ae*Kir2B and *Ae*Kir3 [10]. Thus, VU590 represents the second small-molecule inhibitor of a mosquito Kir1 channel, with a relatively superior efficacy and selectivity in vitro compared to VU573.

### VU573 is an unexpected agonist of *Ae*Kir2B

Given that VU573 inhibits *Ae*Kir1 (Figure 2; [9]) and several mammalian Kir channels [13], we expected this compound to also inhibit *Ae*Kir2B. Thus, we were surprised to find that VU573 activates *Ae*Kir2B (Figure 2). How a pharmacological agent blocks most Kir channels and activates at least one is a mystery. One potential explanation for the dual effects of VU573 is that it interacts with a regulatory or gating domain of the channel that is differentially regulated between *Ae*Kir1 and *Ae*Kir2B. Thus, the binding of VU573 to *Ae*Kir1 could disrupt an interaction with a channel agonist resulting in a lower activity, whereas the binding of VU573 to *Ae*Kir2B could enhance an interaction with this agonist, resulting in a greater activity. Resolving the precise regulatory mechanism behind such dual effects of VU573 will likely require an exhaustive effort, because Kir-channel activity is known to be regulated by a wide variety of intracellular factors including ATP, pH, Na^+^, G proteins, PIP_2_, and phosphorylation [17].

The unexpected effects of VU573 on *Ae*Kir2B highlight yet another functional and/or pharmacological difference between the *Ae*Kir1 and *Ae*Kir2B channels in vitro. We have previously shown that compared to *Ae*Kir2B channels, *Ae*Kir1 channels 1) mediate more robust inward K^+^ currents, 2) are activated by elevated extracellular Na^+^ concentrations, and 3) are unable to mediate inward Cs^+^ currents [10]. Moreover, in the present study we show that VU590 blocks *Ae*Kir1, but not *Ae*Kir2B (Figure 2). Taken together these data indicate that *Ae*Kir1 and *Ae*Kir2B likely play divergent physiological roles under novel mechanisms of regulation within the mosquito cells and tissues in which they reside.

### VU590 kills mosquitoes and impairs their excretory capacity

In the present study, we show that injecting the hemolymph of mosquitoes with VU590, with a small volume and K^+^ load, leads to outright death within 24 h without obvious sub-lethal effects, such as the loss of flight. Importantly, the inactive analog VU608 does not kill mosquitoes (Figure 3B), which suggests that the toxic effects of VU590 are due to the inhibition of *Ae*Kir1. In a previous study, we observed that similar injections of VU573 primarily led to the loss of flight within 24 h, but not death [9].

Notably, the doses of VU590 required to kill mosquitoes (≥ 0.5 nmol; Figure 3A) are appreciably greater than those required of VU573 to impair the flight of mosquitoes (≥ 40 pmol; [9]). However, the less potent effects of VU590 in vivo are consistent with the in vitro data suggesting that VU590 only inhibits *Ae*Kir1, while VU573 modulates the activity of both *Ae*Kir1 and *Ae*Kir2B. *A. aegypti* mosquitoes possess two copies of the *Ae*Kir2B gene: *Ae*Kir2B and *Ae*Kir2B' [10]. We have shown that at least one of these *Ae*Kir2B genes is expressed in the excretory system [10] as well as in the head and thorax/abdomen of adult female mosquitoes [11]. Thus, it is tempting to speculate that the potent impairment of flight by VU573, which is not observed in VU590-treated mosquitoes, is in part due to its ability to activate *Ae*Kir2B channels in the central nervous system and/or flight musculature.

In the present study, we show that treatment of adult female mosquitoes with VU590, but not VU608, leads to abdominal bloating in adult female mosquitoes as well as disruptions to their excretory capacity. These findings suggest that *Ae*Kir1 plays a role in mosquito renal physiology, as we have suggested previously [9], [10]. Moreover, these findings are consistent with our previous study that showed VU573 inhibits urine production in isolated Malpighian tubules and urine excretion in intact adult female mosquitoes, thereby causing abdominal bloating and renal failure [9]. However, VU573 is more effective at inhibiting urine excretion in vivo than VU590 (Figure 4), which may stem from its modulation of both *Ae*Kir1 and *Ae*Kir2B.

In the excretory system, *Ae*Kir1 is expressed in the Malpighian tubules, and *Ae*Kir2B is expressed in both the Malpighian tubules and hindgut [10], [11]. Thus, depending on the specific functional roles of *Ae*Kir1 and *Ae*Kir2B in the 1) production of urine by the tubules, 2) reabsorption of urinary water and ions by the hindgut, and 3) expulsion of urine by the hindgut, the additional activation of *Ae*Kir2B by VU573 may lead to greater inhibitory effects on urine output than just the sole inhibition of *Ae*Kir1 by VU590. A more precise understanding will require immunolocalization studies of the *Ae*Kir1 and *Ae*Kir2B proteins in the tubule and hindgut epithelia, and in vitro physiological studies that compare the effects of VU590 and VU573 on 1) urine production and electrophysiology in isolated Malpighian tubules, and 2) fluid/ion transport and muscle contractions in isolated hindguts.

In conclusion, the present study validates the *Ae*Kir1 channel as an insecticide target and confirms that small molecule modulators of Kir channels offer valuable new active compounds for insecticide development. To date, the only chemicals identified (i.e., VU573 and VU590) are imperfect in that they are more potent inhibitors of mammalian Kir channels than mosquito Kir channels and require injection into the hemolymph to elicit activity in mosquitoes. Thus, two immediate, remaining challenges are to discover small-molecule inhibitors of mosquito Kir channels that 1) exhibit much greater potency on mosquito Kir channels vs. mammalian Kir channels, and 2) permeate the cuticle of mosquitoes.

## Supporting Information

Figure S1 **Time course of in vivo urine excretion in adult female mosquitoes (** ***A. aegypti*** **) following a volume load.** Cumulative volume of urine excreted by mosquitoes every 30 min for 120 min following injection with 900 nl of the vehicle (K^+^-PBS_50_ containing 1.8% DMSO, 0.077% β-cyclodextrane, and 0.008% Solutol). The cumulative volume excreted at 60 min after injection represents ∼95% of the total volume excreted at 120 min. Values are means ± SEM; *n* = 8 trials of 5 mosquitoes.
(TIF)
