# Supplementary figures and images for: Pharmacological Validation of an Inward-Rectifier Potassium (Kir) Channel as an Insecticide Target in the Yellow Fever Mosquito Aedes aegypti
Source: PLoS One. 2014 Jun 24;9(6):e100700. doi: 10.1371/journal.pone.0100700 (PMC4069099; doi:10.1371/journal.pone.0100700)

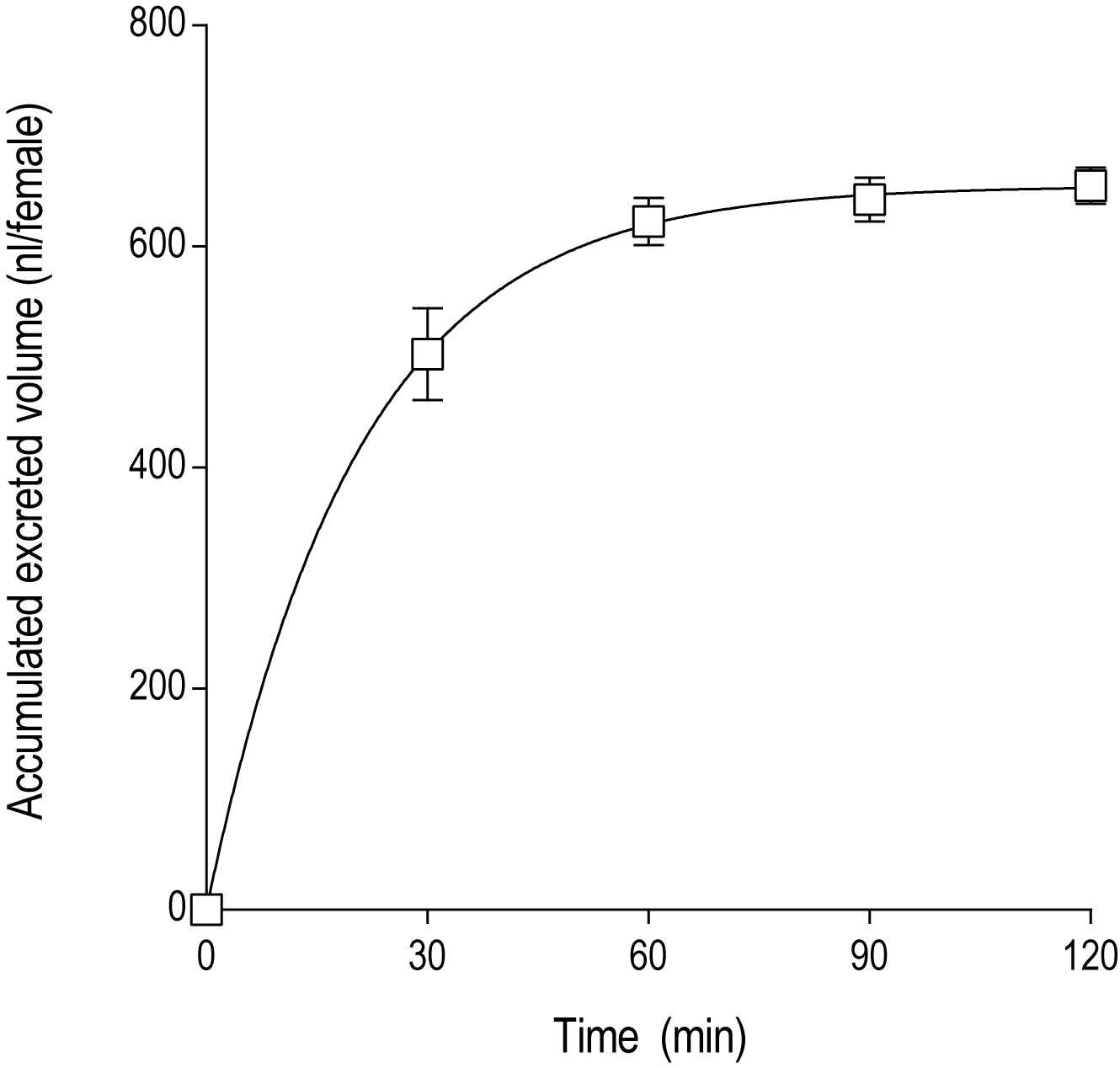

Supplement: Figure S1 — Time course of in vivo urine excretion in adult female mosquitoes ( A. aegypti ) following a volume load. Cumulative volume of urine excreted by mosquitoes every 30 min for 120 min following injection with 900 nl of the vehicle (K+-PBS50 containing 1.8% DMSO, 0.077% β-cyclodextrane, and 0.008% Solutol). The cumulative volume excreted at 60 min after injection represents ∼95% of the total volume excreted at 120 min. Values are means ± SEM; n = 8 trials of 5 mosquitoes. (TIF) [file pone.0100700.s001.tif]
